# Increased prevalence of cardiovascular disease in idiopathic normal pressure hydrocephalus patients compared to a population-based cohort from the HUNT3 survey

**DOI:** 10.1186/2045-8118-11-19

**Published:** 2014-08-19

**Authors:** Per Kristian Eide, Are Hugo Pripp

**Affiliations:** 1 Department of Neurosurgery, Oslo University Hospital - Rikshospitalet, PB 4950 Nydalen, 0424 Oslo, Norway; 2 Faculty of Medicine, University of Oslo, Oslo, Norway; 3 Department of Biostatistics, Epidemiology and Health Economics, Oslo University Hospital, Oslo, Norway

**Keywords:** Idiopathic normal pressure hydrocephalus, Cardiovascular disease, The HUNT3 Survey, General population, Arterial hypertension, Angina pectoris, Cardiac infarction, Diabetes

## Abstract

**Background:**

Idiopathic normal pressure hydrocephalus (iNPH) is one of few types of dementia that can be treated with shunt surgery and cerebrospinal fluid (CSF) diversion. It is frequently present with cerebral vasculopathy; however, how the prevalence of cardiovascular disease compares between iNPH patients and the general population has not yet been established. Therefore, a case–control study was performed to examine whether the prevalence of cardiovascular disease (arterial hypertension, angina pectoris, cardiac infarction, and diabetes) was different in 440 iNPH patients, when compared to 43,387 participants of the Nord-Trøndelag Health 3 Survey (The HUNT3 Survey), which was considered as the general control population.

**Findings:**

In iNPH patients aged 35–70 years, we found increased prevalence for arterial hypertension (males), angina pectoris (females and males), and cardiac infarction (males), as compared with the HUNT3 control group with significant odds ratio estimates. In addition, the prevalence of diabetes was increased in both age groups 35–70 years (males) and 70–90 years (females and males).

**Conclusions:**

The data show significantly increased prevalence of cardiovascular disease iNPH patients, which provide evidence that cardiovascular disease is involved as an exposure in the development of iNPH.

## Findings

### Introduction

The clinical entity idiopathic normal pressure hydrocephalus (iNPH) is characterized by dementia, gait ataxia, urinary incontinence and enlarged cerebral ventricles [1]. Even though the disease was described about 50 years ago [2], its cause remains unknown.

Several lines of evidence suggest an association between cardiovascular disease as an exposure and risk factor in the development of iNPH [3-8]. However, the studies have included a small number of patients and hospital-based control groups. Thus, it was recently pointed out that further studies are needed to clarify the association between iNPH and cardiovascular risk factors, preferably including population-based controls [9].

This study was undertaken to examine whether the occurrence of cardiovascular disease in iNPH patients is higher than in the general population. In Norway a population based public health study, The Nord-Trøndelag Health Study (HUNT), has run since 1984. More than 50,000 individuals participated in The HUNT3 (2006–2008) survey. We compared the occurrence of cardiovascular disease in iNPH patients managed within our department 2002–2011 with the HUNT3 cohort.

## Materials and methods

The study was approved by The Regional Committee for Medical and Health Research Ethics (REK) of Health Region South-East, Norway (2012/1180), and by Oslo University Hospital (2011/6692), Oslo, Norway. A case–control study design was used.

### iNPH patients

The patient material included patients managed for probable or possible iNPH within the Department of Neurosurgery, Oslo University Hospital, Rikshospitalet, during 10-year period 2002 to 2011. The diagnosis of iNPH was based on clinical neurological examination, radiological assessment of ventricular size using computed tomography (CT) or MRI. Normal intracranial pressure (ICP) was documented by over-night ICP monitoring. The clinical neurological examination revealed 2–3 of the triad of gait ataxia, urinary incontinence and dementia, increased ventricular size revealed by CT or MRI. The diagnostic criteria of probable and possible iNPH has been described previously [10]. It was beyond the scope of this study to differentiate the probable and possible iNPH sub-groups.

### Population based HUNT3 cohort

Data from The HUNT3 Survey was used as an estimate of the control population. During the period 2006–2008, all inhabitants in the county of Nord-Trøndelag, Norway, aged 20 years and older were invited to participate in a general health study, named *Nord-Trøndelag Health Study* 3 (The HUNT3 Survey; http://www.ntnu.no/hunt). This study included physical examinations, blood samples and questionnaires that covered demographic characteristics, somatic illnesses, somatic and mental symptoms, medications, lifestyle and health-related behavior. The population of Nord-Trøndelag County is stable and homogenous with less than 3% non-Caucasians, and is representative for Norway in general, though not containing any large cities.

### The presence of cardiovascular disease as a risk factor

This was defined in the same way in the iNPH and HUNT3 cohorts. For the iNPH patients (i.e. cases), it was either reported by the referring doctor/neurologist, and/or by the patient or his/her relatives. For the participants in The HUNT3 Survey (i.e. controls), it was based on self-reported cardiovascular disease in a standardized questionnaire. The patients/relatives answered the following questions:

Do you take or have you taken medication for high blood pressure?

Have you had or do you have any of the following: angina pectoris (chest pain)?

Have you had or do you have any of the following: myocardial infarction (heart attack)?

Have you had or do you have any of the following: diabetes?

### Data analysis

Descriptive statistics are mean (standard deviation) or number of patients (percentage) and difference between groups assessed with student t-test or chi-square tests for crosstabs if not otherwise stated. Exposure odds ratios (OR) with 95% confidence intervals (95CI) and *p*-values for the cardiovascular diseases were calculated. To take into account both the confounding and modifying effects of age and sex, a stratified analysis on both sex and age at a cut-off of 70 years, was conducted. The odds ratios for the resulting four stratified groups are presented. Effect of residual differences in age distribution between cases and controls within these stratified groups were adjusted for using logistic regression by including age as a continuous independent variable. BMI as exposure was examined as a continuous variable and the resulting odds ratio assessed with binary logistic regression. Statistical significance was accepted at the 0.05 level. All statistical analysis was performed using the SPSS software version 20 (IBM Corporation, Armonk, NY).

## Results

Table 1 presents demographic data of the 440 iNPH patients and the 43,387 individuals of the HUNT3 cohort, 35–90 years of age. The subjects were stratified into those aged 35–70 years and 70–90 years, analyzed separately for female and male subjects, and adjusted for age. For the 35–70 years group, the mean age was 61 and 53 years for iNPH and HUNT3 cohorts, respectively, while mean age was 77 years for both iNPH and HUNT3 cohorts of the 70–90 years age groups.

**Table 1:** Demographic data of iNPH cases/HUNT-3 cohort aged 35–90 years

	**iNPH**	**HUNT3**	***p*-value**
N	440	43,387	
Gender (F/M)	220/220	23,372/20,015	NS
Mean age (±std)	70.7 ± 9.8	57.3 ± 12.9	<0.001
Arterial hypertension (N/%)	184 (41.8%)	10,441 (24.1%)	<0.001
Angina pectoris (N/%)	49 (11.1%)	1,769 (4.1%)	<0.001
Cardiac infarction (N/%)	41 (9.3%)	1,625 (3.7%)	<0.001
Diabetes mellitus II (N/%)	69 (15.7%)	2,180 (5.0%)	<0.001
Height (cm)	171.4 ± 8.9	170.3 ± 9.2	NS
Weight (kg)	76.4 ± 15.5	79.8 ± 15.0	<0.001
BMI (kg/m^2^)	26.0 ± 4.6	27.4 ± 4.3	<0.001

iNPH = idiopathic normal pressure hydrocephalus; ^4^HUNT3 = The HUNT3 Survey. NS = non-significant.

Tables 2, 3, 4 and 5 present the prevalence of arterial hypertension, angina pectoris, cardiac infarction, and diabetes. With reference to the age-adjusted ORs (Tables 2, 3, 4 and 5, right column), the iNPH patients 35–70 years of age presented with significantly increased occurrence of arterial hypertension (males; Table 2), angina pectoris (females and males; Table 3), cardiac infarction (males; Table 4). The prevalence of diabetes was increased in both age groups 35–70 years (males) and 70–90 years (females and males; Table 5).

**Table 2:** Prevalence of arterial hypertension according to gender and age-group in iNPH cases/HUNT3 cohort

				**Arterial hypertension**		**Crude estimate**	**Age-adjusted estimate**
	**Age (years)**	**Gender**	**Total**	**Yes**	**No**	**OR (95% CI), *p*-value**	**OR (95% CI), *p*-value**
iNPH	≥ 35 - 70	Female	95	32 (33.7%)	63 (66.3%)	2.4 (1.5 – 3.6), <0.001	1.4 (0.9 – 2.1), NS
HUNT3	≥ 35 - 70	Female	18,988	3,353 (17.7%)	15,635 (82.3%)		
iNPH	≥ 35 - 70	Male	81	40 (49.4%)	41 (50.6%)	4.1 (2.7 – 6.4), <0.001	2.2 (1.4 – 3.5), <0.001
HUNT3	≥ 35 - 70	Male	16,425	3,140 (19.1%)	13,285 (80.9%)		
iNPH	≥70 - 90	Female	125	58 (46.4%)	67 (53.6%)	0.8 (0.6 – 1.2), NS	0.9 (0.6 – 1.2), NS
HUNT3	≥70 - 90	Female	4,384	2,215 (50.5%)	2,169 (49.5%)		
iNPH	≥70 - 90	Male	139	54 (38.8%)	85 (61.2%)	0.7 (0.5 – 1.0), 0.030	0.7 (0.5 – 1.0), 0.030
HUNT3	≥70 - 90	Male	3,590	1,733 (48.3%)	1,857 (51.7%)		

iNPH = idiopathic normal pressure hydrocephalus; ^4^HUNT3 = The HUNT3 Survey; OR = odds ratio; CI = confidence interval. Data presented as numbers (percentages in parenthesis). NS = non-significant.

**Table 3:** Prevalence of angina pectoris according to gender and age-group in iNPH cases/HUNT3 cohort

				**Angina pectoris**		**Crude estimate**	**Age-adjusted estimate**
	**Age (years)**	**Gender**	**Total**	**Yes**	**No**	**OR (95% CI), *p*-value**	**OR (95% CI), *p*-value**
iNPH	≥ 35 - 70	Female	95	6 (6.3%)	89 (93.7%)	6.0 (2.6 – 13.8), <0.001	3.3 (1.4 – 7.7), 0.006
HUNT3	≥ 35 - 70	Female	18,988	212 (1.1%)	18,776 (98.9%)		
iNPH	≥ 35 - 70	Male	81	10 (12.3%)	71 (87.7%)	4.4 (2.4 – 8.5), <0.001	2.2 (1.1 – 4.3), 0.023
HUNT3	≥ 35 - 70	Male	16,425	514 (3.1%)	15,911 (96.9%)		
iNPH	≥70 - 90	Female	125	10 (8.0%)	115 (92.0%)	0.7 (0.4 – 1.4), NS	0.7 (0.4 – 1.4), NS
HUNT3	≥70 - 90	Female	4,384	468 (10.7%)	3,916 (89.3%)		
iNPH	≥70 - 90	Male	139	23 (16.5%)	116 (83.5%)	1.0 (0.7 – 1.6), NS	1.0 (0.7 – 1.6), NS
HUNT3	≥70 - 90	Male	3,590	575 (16.0%)	3,015 (84.0%)		

iNPH = idiopathic normal pressure hydrocephalus; ^4^HUNT3 = The HUNT3 Survey; OR = odds ratio; CI = confidence interval. Data presented as numbers (percentages in parenthesis). NS = non-significant.

**Table 4:** Prevalence of cardiac infarction according to gender and age-group in iNPH cases/HUNT3 cohort

				**Cardiac infarction**		**Crude estimate**	**Age-adjusted estimate**
	**Age (years)**	**Gender**	**Total**	**Yes**	**No**	**OR (95% CI), *p*-value**	**OR (95% CI), *p*-value**
iNPH	≥ 35 - 70	Female	95	2 (2.1%)	93 (97.9%)	2.8 (0.7 – 11.6), NS	1.5 (0.4 – 6.4), NS
HUNT3	≥ 35 - 70	Female	18,988	143 (0.8%)	18,845 (99.2%)		
iNPH	≥ 35 - 70	Male	81	13 (16.0%)	68 (84.0%)	5.0 (2.8 – 9.1), <0.001	2.6 (1.4 – 4.7), 0.002
HUNT3	≥ 35 - 70	Male	16,425	604 (3.7%)	15,821 (96.3%)		
iNPH	≥70 - 90	Female	125	7 (5.6%)	118 (94.4%)	0.8 (0.64 – 1.8), .NS	0.8 (0.4 – 1.8), NS
HUNT3	≥70 - 90	Female	4,384	298 (6.8%)	4,086 (93.2%)		
iNPH	≥70 - 90	Male	139	19 (13.7%)	120 (86.3%)	0.8 (0.5 – 1.3), NS	0.8 (0.5 – 1.3), NS
HUNT3	≥70 - 90	Male	3,590	580 (16.2%)	3,010 (83.8%)		

INPH = idiopathic normal pressure hydrocephalus; ^4^HUNT3 = The HUNT3 Survey; OR = odds ratio; CI = confidence interval. Data presented as numbers (percentages in parenthesis). NS = non-significant.

**Table 5:** Prevalence of diabetes according to gender and age-group in iNPH cases/HUNT3 cohort

				**Diabetes**		**Crude estimate**	**Age-adjusted estimate**
	**Age (years)**	**Gender**	**Total**	**Yes**	**No**	**OR (95% CI), *p*-value**	**OR (95% CI), *p*-value**
iNPH	≥ 35 - 70	Female	95	6 (6.3%)	89 (93.7%)	2.0 (0.9 – 4.5), NS	1.3 (0.6 – 2.9), NS
HUNT3	≥ 35 - 70	Female	18,988	635 (3.3%)	18,353 (96.7%)		
iNPH	≥ 35 - 70	Male	81	20 (24.7%)	61 (75.3%)	6.7 (4.0 – 11.2), <0.001	4.0 (2.4 – 6.8), <0.001
HUNT3	≥ 35 - 70	Male	16,425	769 (4.7%)	15,656 (95.3%)		
iNPH	≥70 - 90	Female	125	21 (16.8%)	104 (83.2%)	2.0 (1.2 – 3.1), 0.007	2.0 (1.2 – 3.2), 0.006
HUNT3	≥70 - 90	Female	4,384	413 (9.4%)	3,971 (90.6%)		
iNPH	≥70 - 90	Male	139	22 (15.8%)	117 (84.2%)	1.7 (1.1 – 2.7), .031	1.7 (1.1 – 2.7), .031
HUNT3	≥70 - 90	Male	3,590	363 (10.1%)	3,227 (89.9%)		

INPH = idiopathic normal pressure hydrocephalus; ^4^HUNT3 = The HUNT3 Survey; OR = odds ratio; CI = confidence interval. Data presented as numbers (percentages in parenthesis). NS = non-significant.

In the group of 440 iNPH patients, 289 were manged with shunt surgery while 151 were managed conservatively. Notably, the ORs for arterial hypertension, angina pectoris, cardiac infarction and diabetes were comparable between iNPH patients in the surgery group (n = 289) and the total cohort (n = 440; data not shown).

## Discussion

The main observation here is that the prevalence of arterial hypertension, angina pectoris, cardiac infarction and diabetes was significantly increased in iNPH patients as compared with the general population, represented by the HUNT3 cohort.

O’Connell was the first to suggest that cerebrovascular disease and white matter lesions could result in hydrocephalus [11]. Since iNPH was described in 1965, only a few studies incorporating a rather small number of patients have explored the occurrence of cardiovascular disease in iNPH. Hence, arterial hypertension was seen in 14 of 19 (74%) iNPH patients while in 38 of 142 (27%) control subjects [7]. In another study, arterial hypertension, diabetes, and ischemic heart disease was more frequent in a group of 17 iNPH patients than in 51 control subjects [8]. Furthermore, Krauss *et al.*[6] reported arterial hypertension in 54/65 (83%) iNPH patients but only in 25/70 (36%) control subjects. Regarding diabetes, one study reported diabetes in 17/33 (52%) iNPH cases, as compared to 4/33 (12%) age-matched controls [12]. While the studies referred to used small hospital-based control groups, the present study is the first study to compare cardiovascular disease against a general population-based cohort in iNPH patients.

The need for population-based studies relates to the fact that the occurrence of cardiovascular disease highly depends on age, race, gender and geographic location. For example, regarding arterial hypertension, Wolf-Maier *et al.*[13] showed the great variance of the prevalence of arterial hypertension between and within continents. While about 28% of the population in North-America had high blood pressure (>140/90), the prevalence of arterial hypertension in Europe was overall 44%. Great variation was even seen within Europe; Germany had the highest frequency (55%) and Italy the lowest (38%). We decided to use data from a big population based study (The HUNT3 Survey) for the comparison of the prevalence of cardiovascular disease. Hence, our control population is the same as the source population for the iNPH patient cohort.

The diagnosis of iNPH is not very well defined. Thus, a major issue has been to precisely define which patients that will respond to shunting, which is the only effective treatment [14]. With regard to determining the prevalence of cardiovascular disease in iNPH, the lack of strict diagnostic criteria represents a challenge. Recently, a classification of iNPH differentiated between probable, possible and unlikely iNPH [10]. Since, the present patients included probable or possible iNPH, we consider the present patient cohort to be a representative cohort of iNPH patients. Moreover, we compared the occurrence of cardiovascular disease and ORs between the total cohort of 440 iNPH patients and those 289 undergoing shunt surgery and found comparable results.

The method used for diagnosing cardiovascular disease can be discussed. One obvious limitation is that the presence of disease is self-reported. Particularly in iNPH patients with variable degree of cognitive failure, this may lead to under-reporting of occurrence of cardiovascular disease, even though relatives answered as well. In the present study cohort, the prevalence of cardiovascular disease was particularly increased in males, and individuals aged 35–70 years. However, the prevalence of angina pectoris and diabetes was also increased in females and for diabetes in the age group 70–90 years. Accordingly, cardiovascular disease as an exposure seems to affect both genders and all age groups from 35–90 years.

It is presently not clear how vascular pathology such as atherosclerosis affect CSF homeostasis. The cerebral water compartments are closely linked to the cerebrovascular system [15]. Recently the importance of paravascular water exchange in the brain was described [16,17]. Hence, further studies are needed to understand how vascular pathology affects cerebral water exchange.

## Conclusions

The data show significantly increased prevalence of cardiovascular disease in iNPH patients, which provide evidence that cardiovascular disease is involved as an exposure in the development of iNPH.

## Abbreviations

BMI: Body mass index; CI: Confidence interval; CSF: Cerebrospinal fluid; CT: Computer tomography; ICP: Intracranial pressure; HUNT3: The HUNT3 Survey; iNPH: Idiopathic normal pressure hydrocephalus; MRI: Magnetic resonance imaging; OR: Odds ratio.

## Competing interests

The authors declare that they have no competing interests.

## Authors’ contributions

PKE contributed to the conception and design of the work, the acquisition, analysis and interpretation of the data, and the bulk of the drafting of the article, while AHP contributed with statistical analysis and editing of the manuscript. Both authors agree to be accountable for all aspects of the work in ensuring that questions related to the accuracy or integrity of any part of the work are appropriately investigated and resolved. Both authors read and approved the final manuscript.
